# Elderly Onset Sarcoidosis: A Case Report

**DOI:** 10.7759/cureus.20443

**Published:** 2021-12-15

**Authors:** Lintu Ramachandran, Saagar Pamulapati, Aisha Barlas, Ammar Aqeel

**Affiliations:** 1 Internal Medicine, Javon Bea Hospital, Rockford, USA

**Keywords:** eos, hypercalcemia, sarcoidosis, late onset sarcoidosis, elderly onset sarcoidosis

## Abstract

Sarcoidosis is a multi-organ autoimmune disease that affects females more than males. While primarily considered as a disease of the young, very few cases of sarcoidosis have been reported in patients over 65 years old. We report the case of sarcoidosis in an 80-year-old female and ultimately died from sarcoidosis-related complications. We review the literature and highlight key differences in elderly onset sarcoidosis when compared to the general population. We also advise physicians to have a high index of suspicion for sarcoidosis in the elderly who present with hypercalcemia and abnormal findings on chest imaging.

## Introduction

Sarcoidosis is a granulomatous disease that can affect multiple organ systems including the heart, lungs, liver, and lymph nodes. Sarcoidosis is commonly seen in young adults, especially females [1]. The prevalence of sarcoidosis varies largely based on location. In Asian countries, such as Taiwan and South Korea, the estimated prevalence is 1-5 per 100,000 while Sweden and Canada have a significantly higher prevalence of 140-160 per 100,000 [2]. Elderly onset sarcoidosis (EOS), defined as sarcoidosis diagnosed in patients ≥65 years old, is furthermore rare and often has a worse prognosis at onset [3]. We present the case of a patient who presented with nonspecific symptoms and was diagnosed with sarcoidosis at the age of 80 and subsequently died from sarcoidosis.

## Case presentation

**Figure 1:**
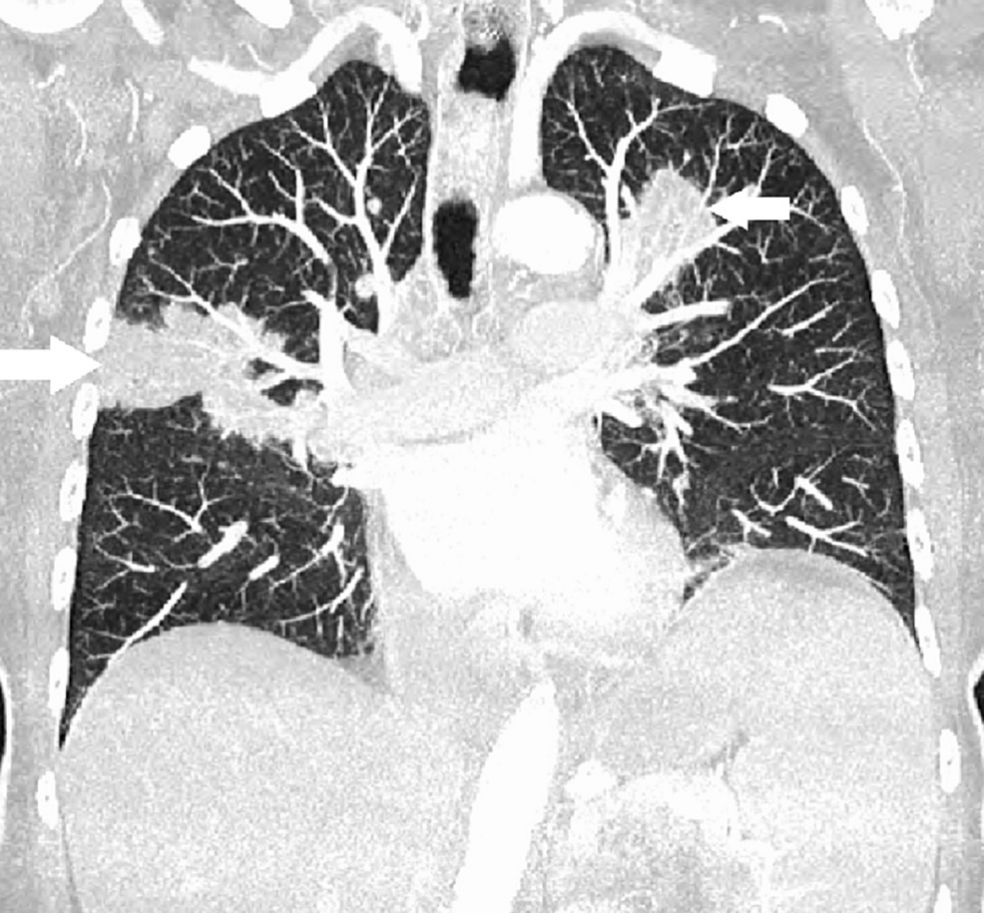
Coronal slice of CT chest indicating the bilateral pulmonary lesions/infiltrates (white arrows)

An 80-year-old Caucasian female with a past medical history of type 2 diabetes, hypertension, and dyslipidemia presented with complaints of fatigue, generalized weakness with associated cough, and poor appetite. Her surgical history included a complete hysterectomy at age 50 but was otherwise unremarkable. Her family history was negative for any autoimmune diseases. She denied any tobacco, alcohol use, or illicit drug use. She had always lived in the United States and denied any recent travel history. Her vitals and physical exam were unremarkable. A complete blood count was normal. However, a basic metabolic panel showed hypercalcemia of 13.1 mg/dL. BUN and creatinine were both normal at 15 mg/dL and 1.0 mg/dL, respectively. Vitamin D level and phosphorus levels were normal at 37 ng/mL and 2.9 mg/dL, respectively. Ionized calcium was found to be elevated at 1.41 mmol/L. Angiotensin-converting enzyme level was elevated at 95 U/L. Chest x-ray showed bilateral hilar lymphadenopathy. A chest x-ray done a year prior for upper respiratory infection and suspected pneumonia did not show any lymphadenopathy. Subsequent chest CT showed multiple mediastinal pulmonary masses, mediastinal lymphadenopathy, bilateral infiltrates, and sclerotic lesions in T2-T7 vertebrae with small lytic foci, concerning metastatic disease. A coronal slice of the CT Chest is shown in Figure 1.

**Figure 2:**
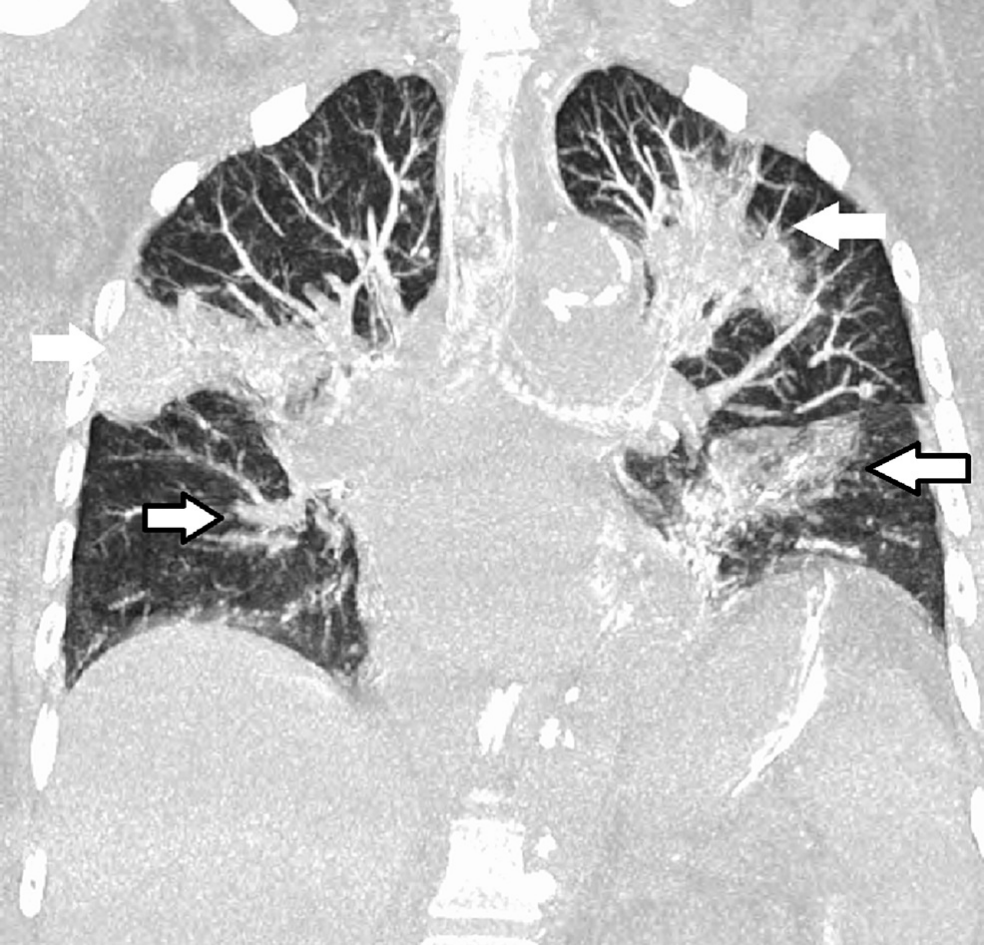
Coronal CT chest showing worsening bilateral infiltrates (white arrows) and new bilateral infiltrates (white arrows with black outline)

CT abdomen was negative for any lesions or lymphadenopathy. A CT-guided biopsy of the lung mass was done which revealed non-caseating granulomas with no evidence of malignancy or infectious process. Acid-fast bacilli staining was negative. She was diagnosed with sarcoidosis considering the constellation of hypercalcemia, lung/bone involvement, and biopsy results. The patient received a six-week course of prednisone, 40mg daily, with a marginal improvement of symptoms. Hypercalcemia also improved to 9.9 mg/dL. The patient was discharged to a rehab facility. She initially improved clinically, but then began to complain again of persistent generalized weakness and worsening dyspnea. She was emergently brought to the ED again and repeat CT imaging showed diffuse worsening of bilateral lung lesions, as well as new lesions in T7 and T11 vertebrae (Figure 2).

The patient's respiratory status progressively declined over her hospital course. Patient and family elected for do not resuscitate/do not intubate (DNR/DNI) and comfort-focused measures only. After five days of hospital stay, the patient passed away.

## Discussion

Sarcoidosis is more common in females and typically has a peak age onset of 20-40 years [4]. There have however been reported bimodal distribution patterns of sarcoidosis with a second peak at age 50 [5]. EOS is rare and there are limited studies that discuss the incidence and prevalence of EOS. One study approximated that 30% of all sarcoidosis cases occur in the elderly [6]. A retrospective study analyzed data from 668 consecutive patients with sarcoidosis over the course of 42 years and found the prevalence of EOS to be 7% [7]. Sarcoidosis at age 80 is furthermore rare. The oldest reported case was that of sarcoidosis at age 81, even though the aforementioned patient had symptoms including heart failure and epistaxis at least since age 79 [8]. Our patient did not have any symptoms of heart failure, renal involvement, or any other sarcoidosis-associated symptoms prior to this admission.

There also seems to be some variation in presentation and lab findings associated with EOS. Fatigue, uveitis, and Chest x-ray findings were commonly seen in EOS. EOS patients also had more comorbidities compared to the young patients with sarcoidosis [9]. Even though erythema nodosum was rarely seen in patients with EOS, other nonspecific sarcoidosis-associated cutaneous lesions were frequently noted in EOS [10]. Lab abnormalities such as hypercalcemia and elevated angiotensin-converting enzyme levels were also more common in EOS [11]. Our patient reported fatigue as her primary chief complaint and had lymphadenopathy on a chest x-ray during her admission. She also had hypercalcemia and elevated angiotensin-converting enzyme levels. However, our patient did not have any skin lesions noted on a thorough physical exam and did not report any uveitis symptoms.

Sarcoidosis presenting in the elderly also seems to be associated with a worse prognosis [10,12]. Hypercalcemia, which was more common in EOS as mentioned earlier, was found to be associated with poorer outcomes in all patients with sarcoidosis [12]. Several theories exist to explain the mortality in the elderly with sarcoidosis. One theory explains that older patients tend to generally have more comorbidities and as such have poorer prognoses [13]. Another theory attributes the high mortality rate of EOS to higher rates of sarcoidosis-related pulmonary fibrosis in the elderly and high fatality rates in sarcoidosis-associated pulmonary fibrosis patients [14]. Our patient had findings of bilateral infiltrates that progressively worsened on subsequent imaging as noted in Figures 1, 2. Our patient's hypoxic respiratory failure from sarcoidosis eventually led to her demise.

## Conclusions

In conclusion, we present the case of an elderly patient who presented with fatigue, weakness, hypercalcemia, and was diagnosed with sarcoidosis. She ultimately passed away from sarcoidosis-related complications. Physicians should have a high index of suspicion for sarcoidosis in elderly patients presenting with hypercalcemia, as EOS can be associated with poor overall prognosis. Knowing the differences in features of EOS and being able to recognize them early can have potential lifesaving implications.
